# Supplementary material for: COZOID: contact zone identifier for visual analysis of protein-protein interactions
Source: BMC Bioinformatics. 2018 Apr 6;19:125. doi: 10.1186/s12859-018-2113-6 (PMC5889581; doi:10.1186/s12859-018-2113-6)
Supplement: Supplementary file 2 — Software build. Executable binary file of the software tool. (ZIP 118,784 kb) [file 12859_2018_2113_MOESM2_ESM.zip › cozoid/java/docs/beansbinding-1.2.1-doc.zip!/index-files/index-15.html]

O-Index (Beans Binding)


---


|  |  |  |
| --- | --- | --- |
| **Overview** \| Package \| Class \| Use \| **Tree** \| **Deprecated** \| **Index** \| **Help** | |  |
| **PREV LETTER**   **NEXT LETTER** | **FRAMES**    **NO FRAMES**     **All Classes** |  |


A B C D E F G H I J K L M N O P Q R S T U V W 

---


## **O**

**ObjectProperty**<S> - Class in org.jdesktop.beansbinding: An immutable, read-only, `Property` implementation whose `getValue` method returns the source object that it is given. **ObservableCollections** - Class in org.jdesktop.observablecollections: `ObservableCollections` provides factory methods for creating observable lists and maps. **ObservableCollections()** - Constructor for class org.jdesktop.observablecollections.ObservableCollections: **ObservableCollections.ObservableListHelper**<E> - Class in org.jdesktop.observablecollections: `ObservableListHelper` is created by `observableListHelper`, and useful when changes to individual elements of the list can be tracked. **observableList(List<E>)** - Static method in class org.jdesktop.observablecollections.ObservableCollections: Creates and returns an `ObservableList` wrapping the supplied `List`. **ObservableList**<E> - Interface in org.jdesktop.observablecollections: A `List` that notifies listeners of changes. **observableListHelper(List<E>)** - Static method in class org.jdesktop.observablecollections.ObservableCollections: Creates and returns an `ObservableListHelper` wrapping the supplied `List`. **ObservableListListener** - Interface in org.jdesktop.observablecollections: Notification types from an `ObservableList`. **observableMap(Map<K, V>)** - Static method in class org.jdesktop.observablecollections.ObservableCollections: Creates and returns an `ObservableMap` wrapping the supplied `Map`. **ObservableMap**<K,V> - Interface in org.jdesktop.observablecollections: A `Map` that notifies listeners of changes to the `Map`. **ObservableMapListener** - Interface in org.jdesktop.observablecollections: Notification types from an `ObservableMap`. **Or()** - Method in class org.jdesktop.el.impl.parser.ELParser: **OR0** - Static variable in interface org.jdesktop.el.impl.parser.ELParserConstants: **OR1** - Static variable in interface org.jdesktop.el.impl.parser.ELParserConstants: **org.jdesktop.beansbinding** - package org.jdesktop.beansbinding: Provides support for defining properties and creating bindings between sets of two properties. **org.jdesktop.beansbinding.ext** - package org.jdesktop.beansbinding.ext: Provides support for customizing Beans Binding, and for determining the synthetic properties available for binding. **org.jdesktop.el** - package org.jdesktop.el: Customized version of EL for Beans Binding; not for general use. **org.jdesktop.el.impl** - package org.jdesktop.el.impl: Customized version of EL for Beans Binding; not for general use. **org.jdesktop.el.impl.lang** - package org.jdesktop.el.impl.lang: Customized version of EL for Beans Binding; not for general use. **org.jdesktop.el.impl.parser** - package org.jdesktop.el.impl.parser: Customized version of EL for Beans Binding; not for general use. **org.jdesktop.el.impl.util** - package org.jdesktop.el.impl.util: Customized version of EL for Beans Binding; not for general use. **org.jdesktop.observablecollections** - package org.jdesktop.observablecollections: Provides support for observing changes to collection classes. **org.jdesktop.swingbinding** - package org.jdesktop.swingbinding: Provides support for binding to complex Swing components, and documentation on the interesting Swing properties to bind to. **org.jdesktop.swingbinding.adapters** - package org.jdesktop.swingbinding.adapters: Private implementation details for Swing binding. **org.jdesktop.swingbinding.impl** - package org.jdesktop.swingbinding.impl: Private implementation details for Swing binding. **owner** - Variable in class org.jdesktop.el.impl.lang.FunctionMapperImpl.Function

---


|  |  |  |
| --- | --- | --- |
| **Overview** \| Package \| Class \| Use \| **Tree** \| **Deprecated** \| **Index** \| **Help** | |  |
| **PREV LETTER**   **NEXT LETTER** | **FRAMES**    **NO FRAMES**     **All Classes** |  |


A B C D E F G H I J K L M N O P Q R S T U V W 

---
